# Response to Generative AI in Dental Licensing Examinations: Comment

**DOI:** 10.1016/j.identj.2024.02.002

**Published:** 2024-02-25

**Authors:** Reinhard Chun Wang Chau, Khaing Myat Thu, Ollie Yiru Yu, Edward Chin Man Lo, Richard Tai-Chiu Hsung, Walter Yu Hang Lam

**Affiliations:** Faculty of Dentistry, The University of Hong Kong, Hong Kong, China; Faculty of Dentistry, The University of Hong Kong, Hong Kong, China; Department of Computer Science, Hong Kong Chu Hai College, Hong Kong, China; Faculty of Dentistry, The University of Hong Kong, Hong Kong, China; Musketeers Foundation Institute of Data Science, The University of Hong Kong, Hong Kong, China

Dear Editor,

Thank you for allowing us to respond to the letter written on the "Performance of Generative Artificial Intelligence in Dental Licensing Examinations".^1^ We would like to express our gratitude to Hinpetch Daungsupawong and Viroj Wiwanitkit for their interest and comments. We are pleased to address these comments and engage in further discussion regarding the findings.

We agree with the suggestion to expand the evaluation to include domains and question types other than multiple-choice questions (MCQs) to obtain a more comprehensive understanding of Generative Artificial Intelligence (GenAI)’s proficiency, as mentioned in our discussion for future study direction. In this particular study, we chose to use MCQs from selected dental licensing examinations as the measurement metrics because they allowed for an objective assessment of the correctness of GenAI responses. This approach helped avoid potential human errors in analysing the content of answers.^2^^,^^3^ One relevant aspect to consider is Artificial hallucination, which is a phenomenon where the GenAI generates information that appears realistic but is, in fact entirely fabricated without any factual foundation.^4^^,^^5^ However, future research could incorporate open-ended questions and practical scenarios to test GenAI in contexts that require higher-order cognitive skills.^6^

Regarding the degree of difficulty of the MCQs used in the testing, we used questions from two dental licensing examinations that covered a broad range of dental subjects. These examinations are considered benchmarks for evaluating the desired level of expertise following professional training. The passing rates of these examinations are determined by a panel of education and clinical experts, serving as an indicator of the expected performance of human test-takers. Additionally, it is important to acknowledge that human performance can be affected by variables such as test-taking strategies and other subjective elements, which may impact the outcomes.^7^^,^^8^ In future studies, it may be worthwhile to consider integrating psychometric analyses to provide a more nuanced understanding of GenAI's performance in relation to question complexity.^9^

With advancements in neural network architecture, including the inclusion of multimodal functionality, there is an expectation that GenAI's performance will continue to improve, as demonstrated in the current study where the newer version of ChatGPT performs better.^10^^,^^11^ The quality and quantity of the training dataset used to train GenAI also significantly impact its performance. The incorporation of a web-browsing function enables ChatGPT 4.0 to update its database. However, it is uncertain if it can access subscription-based databases, which comprise the majority of dental journals and textbooks. It is anticipated that specialized medical and dental GenAI may be developed in the future, potentially exhibiting better performance than the current general-purpose GenAI. Moreover, the ability of GenAI to interpret and provide feedback on graphical and non-text elements is an area that holds promise for future development.^12^^,^^13^

In line with the results of the current study, ChatGPT has demonstrated the ability to pass the medical, legal, and business examinations.^9^^,^^10^ The authors believe that GenAI will be used by both dental professionals and the general public in the clinical settings as well as dental training and education. We agree that it is crucial to consider the ethical implications of integrating GenAI in clinical and educational settings.^14^ The present study serves as a prompt for discussion about responsible integration, and we advocate for the development of ethical guidelines to ensure that GenAI functions as an aid that complements human judgment rather than replacing it. In this regard, international organizations such as the World Health Organization (WHO)^15^ and the World Dental Federation(FDI)^16^ have made efforts to address the ethical issues in association with AI in healthcare.

Yours Sincerely.

## Conflict of interest

The authors declare that they have no known competing financial interests or personal relationships that could have appeared to influence the work reported in this paper.
